# Overexpression of the *Ginkgo biloba* dihydroflavonol 4-reductase gene *GbDFR6* results in the self-incompatibility-like phenotypes in transgenic tobacco

**DOI:** 10.1080/15592324.2022.2163339

**Published:** 2023-01-11

**Authors:** Ning Zhang, Yang Zhan, Kexin Ding, Lijun Wang, Peng Qi, Wona Ding, Maojun Xu, Jun Ni

**Affiliations:** a Key Laboratory of Hangzhou City for Quality and Safety of Agricultural Products, College of Life and Environmental Sciences, Hangzhou Normal University, Hangzhou, China; b Zhejiang Provincial Key Laboratory for Genetic Improvement and Quality Control of Medicinal Plants, Hangzhou Normal University, Hangzhou, China; c College of Science and Technology, Ningbo University, Ningbo, China

**Keywords:** DFR, flavonoids, Ginkgo, seed, self-incompatibility, tobacco

## Abstract

Although flavonoids play multiple roles in plant growth and development, the involvement in plant self-incompatibility (SI) have not been reported. In this research, the fertility of transgenic tobacco plants overexpressing the *Ginkgo biloba* dihydroflavonol 4-reductase gene, *GbDFR6*, were investigated. To explore the possible physiological defects leading to the failure of embryo development in transgenic tobacco plants, functions of pistils and pollen grains were examined. Transgenic pistils pollinated with pollen grains from another tobacco plants (either transgenic or wild-type), developed full of well-developed seeds. In contrast, in self-pollinated transgenic tobacco plants, pollen-tube growth was arrested in the upper part of the style, and small abnormal seeds developed without fertilization. Although the mechanism remains unclear, our research may provide a valuable method to create SI tobacco plants for breeding.

## Introduction

Anthocyanins are synthesized in a branch of the flavonoid pathway and play multiple functional roles in plants.^1^ Dihydroflavonol 4-reductase (DFR, EC1.1.1.219) undertakes the first committed reaction leading to anthocyanin production, and is a key enzyme regulating the carbon flux direction in anthocyanin production.^2^ The overexpression of *DFR* genes leads to anthocyanin increases and phenotype changes in different transgenic plants, indicating various physiological roles of anthocyanins in plants.^3–6^

Self-incompatibility (SI), which prevents inbreeding, thereby maintaining genetic diversity, exists in approximately 40% of all angiosperm species. A number of divergent SI systems have evolved, and type-1 SI (also called Solanaceae-type SI), which was first identified in Solanaceae, has the broadest taxonomic distribution.^7^ In the type-1 SI system, the *S-locus* is composed of two separate determinant genes, *S-RNase* and *S-locus F-box* (*SLF*). S-RNase is the female determinant and is incorporated into the pollen tubes to function as a cytotoxin that degrades pollen RNA. SLF is the male determinant, which is thought to be involved in the ubiquitin-mediated protein degradation of non-self-S-RNases.^8^ There are also other types of SI systems, which bear no similarity to each other, except the two-gene recognition systems. This suggests that SI evolved independently in different angiosperm species.^7^

The “living fossil” *Ginkgo biloba*, which is among the most popular medicinal plants worldwide, contains a number of secondary metabolites. Among these metabolites, flavonoids are the main bioactive constituents, being responsible for the pharmacological activities of ginkgo leaf extracts.^9^ Although, genes involved in the flavonoid biosynthetic pathway have been continuously investigated, most of the researches focused on the correlation between gene expression levels and flavonoid contents, other phenotypes of transgenic plants overexpressing these genes were rarely reported.^4,10–12^

In previous research, we isolated *DFR* genes in ginkgo and examined their roles in regulating anthocyanin contents in both ginkgo and transgenic tobacco (*Nicotiana tabacum*) plants.^4^ Here, we reported the abnormal fertility of transgenic tobacco plants overexpressing *GbDFR6*, and the transgenic tobacco plants exhibited SI-like phenotypes.

## Materials and methods

### Plant materials, PCR confirmations and growth conditions

The genetic information of transgenic tobacco overexpressing *GbDFR6* has been described previously.^4^ For the comparison of seed sizes, at least 30 seeds of each category were measured and compared using the Student’s *t*-test. To produce transgenic *Arabidopsis* plants overexpressing *GbDFR6*, the wild type was *Arabidopsis thaliana* Columbia-0, and the construct used to create transgenic tobacco was reused. The genotyping and reverse transcription PCR (RT-PCR) confirmation of transgenic plants was performed using the primers 5’-GCTTTGGAAAGCCGACTTGG-3’ and 5’-AACGAGTTGCACCTGCCTTA-3’ for *GbDFR6*, and primers 5’-TGGACTCTGGTGATGGTGTC-3’ and 5’-CCTCCAATCCAAACACTGTA-3’ for the control gene *NtACTIN*. PCR reactions of 30 cycles were performed for both *GbDFR6* and *NtACTIN*. For seed germination, tobacco seeds were sterilized with 75% ethanol and planted in plates containing Murashige and Skoog salts (Gibco, Grand Island, NY, USA) and 1.5% (w/v) agar (Becton Dickinson Vacutainer Systems, Rutherford, NJ, USA). All the plants were grown in a green house under a 16-h light and 8-h dark cycle at a constant temperature (25°C).

## Auxin measurement of developing tobacco seeds

Developing seeds were harvested five days after flowering. At this stage, all the seeds were small and undistinguishable. Seeds were weighted immediately after harvest. Then, these seeds were homogenized in PBS solution (pH 7.4) by hand and centrifuged at 3,000 rpm for 20 min. The supernatant was used in the auxin measurements. Measurements of auxin levels were carried out following the instructions of the Auxin ELISA kit (Jingmei Biotechnology, Jiangsu, China). The auxin levels were measured for six times for wild-type and transgenic seeds respectively, and the results were compared using the Mann-Whitney *U* test.

## Southern blot analysis of transgenic tobacco plants

Southern blot analysis was carried out as previously described.^13^ Briefly, the genomic DNA of transgenic tobacco plants (200 mg flesh tissues for each sample) was extracted following the instructions of a Plant Genomic DNA Kit (TIANGEN, Beijing, China). The purified genomic DNA of transgenic tobacco plants was digested at 37°C for 24 h with restriction enzyme *Eco*R I and *Hind* III, respectively. The digested DNA was separated on 0.8% agarose gel, transferred to Hybond-N^+^ nylon membrane (Amersham Pharmacia) and hybridized over night at 45°C with a ^32^P-dCTP-labeled hygromycin-resistant gene probe. The primers used to amplify the probe are 5’-CGTTATGTTTATCGGCACT-3’ and 5’-TTGGCGACCTCGTATTGG-3’.

## *In vitro* pollen viability assays

For pollen germination, flowers with newly opened anthers were used for germination experiments. The dehisced anthers were dipped onto the surface of agar plates to transfer the pollen grains. The medium for *in vitro* pollen germination contained 10% sucrose, 50 mg/L boric acid and 20 mg/L CaCl_2_. Following the pollen application, dishes were transferred to a growth chamber for 12 h at 25°C. To compare the pollen germination ratios between wild-type and transgenic plants, the germination ratios of ten agar plates per plant type were calculated and compared using using the Student’s *t*-test. For each plate, 200 pollen grains were counted to calculate the germination ratio, and pollen tubes longer than the diameter of the pollen grain were considered as germinated. For the pollen 1,2,3-triphenyl tetrazolium chloride (TTC) staining, freshly harvested pollen was dusted onto a microscope slide with a brush to which four or five drops of stain (0.5% TTC) were added. Then the slide was immediately covered with a coverslip and the edges sealed with nail varnish. The staining was examined after 15–30 min incubation at 40°C.

## *In vivo* pollen tube elongation assay

Blooming flowers of wild-type and transgenic tobacco plants were self pollinated manually and isolated to prevent contamination with other pollen grains. Two days after pollination, pistils were harvested and incubated overnight at 65°C in 1 M KOH. After rinsing with water for 2 min, pistils were stained with 0.1% (w/v) aniline in 0.1 M K_2_HPO_4_ (pH 8.5) for 1 h. Callose in the pollen tubes was visualized using a UV filter on a fluorescent microscope.

## RNA degradation assay and measurement of RNase activity

For the collection of extracellular RNase crude extracts from styles, wild-type and transgenic styles were collected and split into equal segments, respectively, to ensure that the section areas were similar. After washing with water for several times, style tissues were soaked in water and evacuated eight times. After each evacuation, the vent valve was opened to let water permeate into the style tissues. Then, the intercellular washing fluids were harvested by centrifugation at 5,000 rpm for 30 min. For the RNA degradation assay, 5 μl RNA (400 ng/μl), 4 μl RNase free water and 1 μl RNase crude extract were mixed and incubated for 0.5 h at 37°C before agarose gel electrophoresis. Measurements of RNase activity were carried out following the instructions of the RNase ELISA kit (Jingmei Biotechnology, Jiangsu, China). The measurements were performed for six times for wild-type and transgenic plants respectively, and the results were compared using the Mann-Whitney *U* test.

## Results

### *Transgenic tobacco plants overexpressing* GbDFR6 *produce smaller seeds*

**Figure 1.**
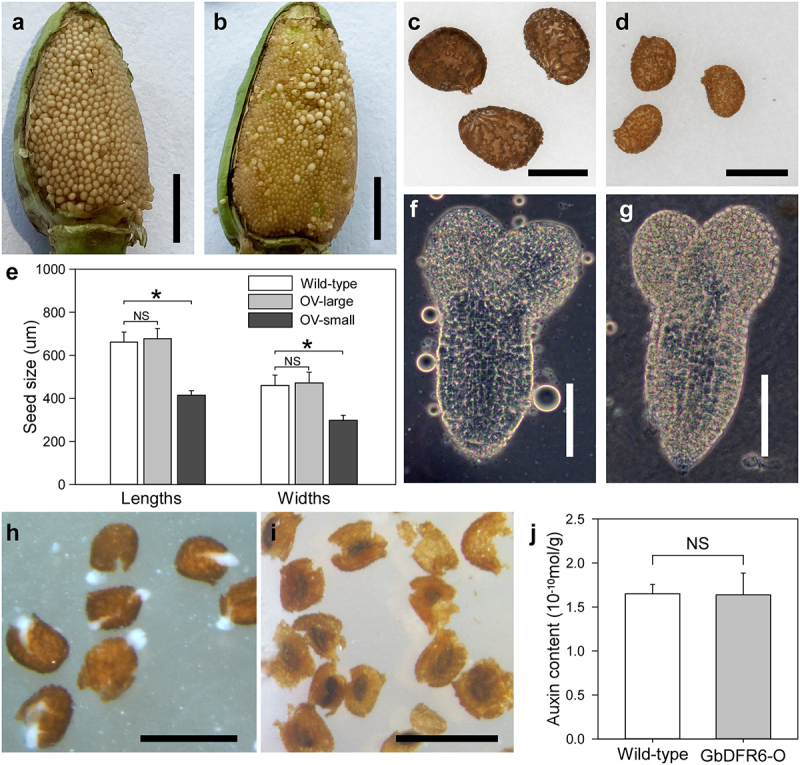
Comparison of seed development between wild-type and transgenic tobacco plants. (a) A wild-type capsule with well-developed seeds. Bar = 0.5 cm. (b) A transgenic capsule with limited well-developed seeds accompanied by a majority of developmentally arrested small seeds. Bar = 0.5 cm. (c) Well-developed wild-type seeds. Bar = 500 μm. (d) Developmentally arrested small seeds. Bar = 500 μm. (e) Comparisons of seed sizes among wild-type seeds, large transgenic seeds and small transgenic seeds. OV, transgenic overexpressing line. Data from independent experiments are shown (means ± SDs; n = 30; Asterisks, significant differences, *P* < .01; NS, not significant, *P* > .05; Student’s *t*-test). (f) A wild-type embryo. Bar = 200 μm. (g) An embryo developed in the transgenic large seed. Bar = 200 μm. (h) Crushed wild-type mature seeds. White tissues are squeezed embryos. Bar = 1 mm. (i) Crushed transgenic small mature seeds. No live tissues were observed. Bar = 1 mm. (j) Comparison of internal auxin contents in developing seeds (mean ± SD; n = 6; NS, not significant, *P* > .05; Mann-Whitney *U* test).

In previous research, transgenic tobacco plants overexpressing *GbDFR* genes showed higher anthocyanin levels than the control.^4^ To further investigate the function of GbDFRs during the reproductive growth stage, the fertility of these transgenic tobacco plants was examined. In contrast to other transgenic tobacco plants, which were similar with wild-type plants, transgenic tobacco plants overexpressing *GbDFR6* showed abnormal phenotype of fertility. In wild-type plants (and transgenic tobacco plants overexpressing *GbDFR4* or *GbDFR5*), well-developed seeds were arranged tightly over whole capsules (Figure 1a). However, in transgenic plants overexpressing *GbDFR6*, the seeds showed developmental arrest, with most being smaller, but limited numbers of well-developed larger seeds remained in the middle regions of the capsules (Figure 1b). Detailed measurements revealed that the lengths and widths of the smaller transgenic seeds were reduced significantly compared with those of wild-type seeds, whereas the larger transgenic seeds were almost the same size as wild-type seeds (Figure 1c–e). In addition, all the large seeds germinated, whereas none of the small seeds germinated. Accordingly, well-developed embryos were observed in the large seeds (Figure 1g), which were identical with wild-type embryos (figure 1f), whereas no traces of embryo tissues were detected in the small seeds. Furthermore, these small seeds were only composed of seed coats, with the inner spaces being empty (Figure 1i). They did not contain the embryo and endosperm tissues that occupied the inner spaces of wild-type seeds (Figure 1h). Thus, embryo development failed in these small seeds. To examine the involvement of auxin in early stage of seed coat development, the auxin contents of wild-type and transgenic seeds were measured and compared. No significant differences in auxin contents were observed between wild-type and transgenic seeds (Figure 1j).

**Figure 2.**
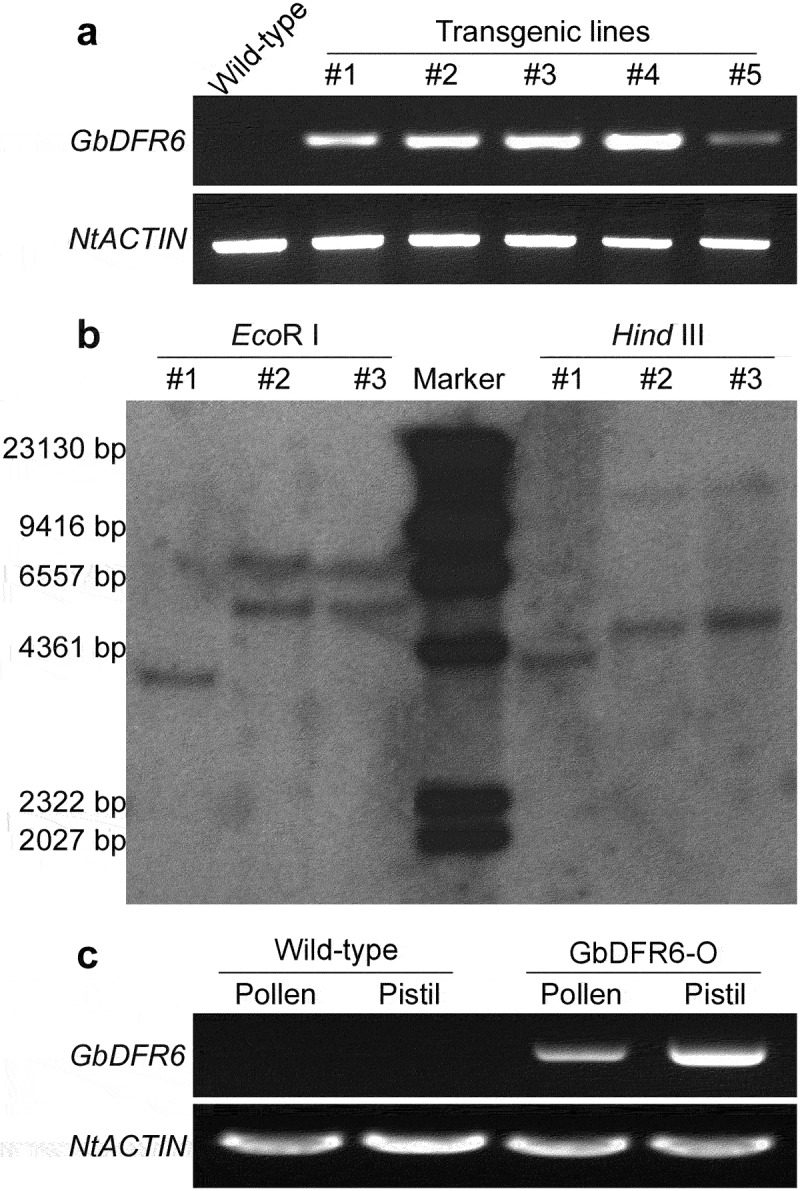
Independence of transgenic tobacco lines. (a) RT-PCR confirmation of *GbDFR6* expression levels in five transgenic tobacco lines. Wild-type tobacco plants do not express *GbDFR6* gene, and transgenic tobacco lines exhibit different *GbDFR6* expression levels. (b) Southern blot analysis of three transgenic tobacco lines. Genomic DNA of three transgenic tobacco lines were digested by *Eco*R I and *Hind* III, respectively. The molecular weights of six marker bands were indicated on the left side. Line #1 was a single copy transgenic line. Line #2 and line #3 were double copy transgenic lines, and they were probably derived from the same line. (c) Expression of *GbDFR6* in the pollen and style of transgenic tobacco plants.

To exclude the possibility of specific insertion in tobacco genome, Southern blot was carried out to examine the independence of these transgenic tobacco lines. First, the *GbDFR6* expression levels were examined in the five transgenic lines exhibiting similar phenotypes (Figure 2a). Then, three of the five lines were selected and two independent lines were observed in the Southern blot analysis (Figure 2b). This proved that the abnormal phenotypes of transgenic tobacco plants were caused by the overexpression of *GbDFR6*, but not the specific insertion in tobacco genome. To confirm the expression of *GbDFR6* in the transgenic reproductive tissues, RNA in pollen and pistil was extracted, and the expression of *GbDFR6* in these tissues were examined. As a result, both of the tissues expressed *GbDFR6* in transgenic tobacco plants (Figure 2c).

## The defect of smaller seeds might be caused by SI

**Figure 3.**
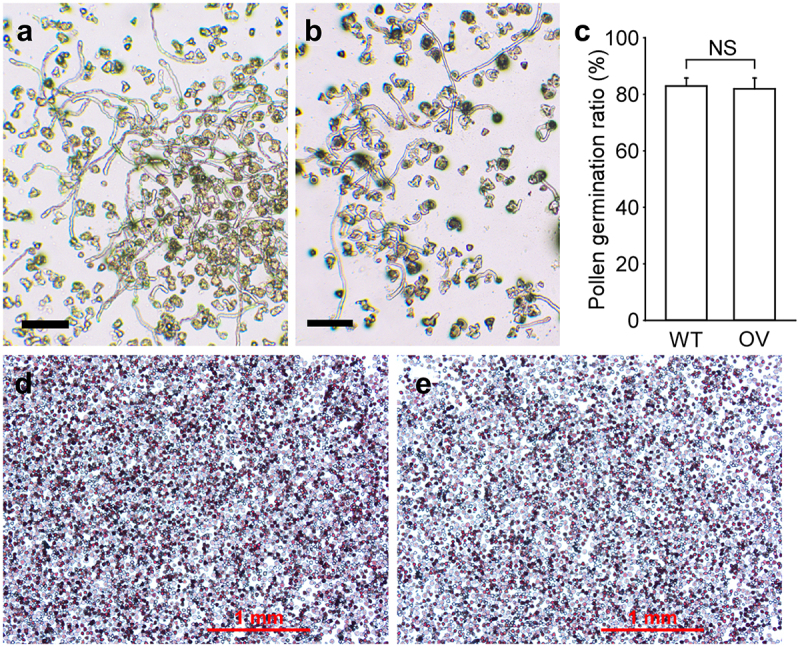
Comparison of wild-type and transgenic pollen viabilities. (a) Germinating pollen grains of wild-type plants. (b) Germinating pollen grains of transgenic plants. Bars = 200 μm. (c) Comparison of pollen germination ratios between wild-type and transgenic plants. OV, transgenic overexpressing line. Data from independent experiments are shown (means ± SDs; n = 10; NS, not significant, *P* > .05; Student’s *t*-test). (d) TTC staining of wild-type pollen grains. (e) TTC staining of transgenic pollen grains. Bars = 1 mm.

To investigate possible physiological defects leading to the failure of embryo development in transgenic tobacco plants, pollen viability was examined using an *in vitro* pollen germination assay. Both wild-type and transgenic pollen grains germinated, and pollen tube elongation was not affected (Figure 3a and b). Furthermore, the pollen germination ratios were similar between wild-type and transgenic plants (Figure 3c). Consistently, TTC staining revealed similar pollen viabilities between wild-type and transgenic pollen grains (Figure 3d and e). To examine the effects of genotypes on embryo development, the seeds from heterozygous and homozygous transgenic plants were compared. The capsules of both genotypes contained similar small/large seed patterns. In addition, the seedlings that developed from the large seeds harvested from heterozygous plants had both wild-type and transgenic genotypes (Fig S1). Thus, the small abnormal seeds in transgenic tobacco plants were not the result of different pollen viability levels or genotypes.

**Figure 4.**
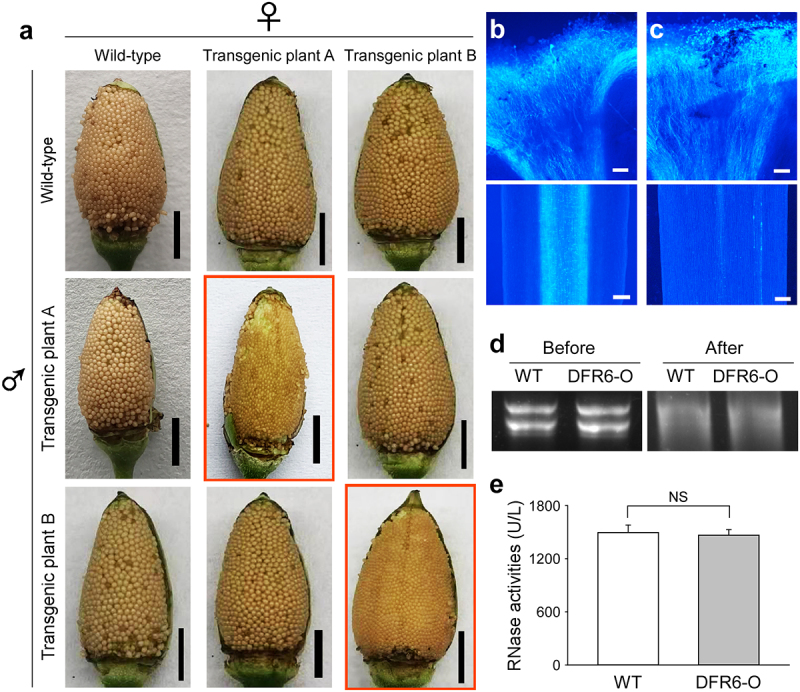
Self-incompatibility of transgenic tobacco plants. (a) Cross-breeding results of different genotypes. Transgenic plant A and transgenic plant B represent different transgenic plants. Self-pollinated results are indicated by red frames. Bar = 500 μm. (b) Fluorescent images of aniline blue-stained wild-type pistils after self pollination. Top, on the stigma. Bottom, in the middle region of the pistil. Bar = 0.4 mm. (c) Fluorescent images of aniline blue-stained transgenic pistils after self pollination. Top, on the stigma. Bottom, in the middle region of the pistil. Bar = 0.4 mm. (d) RNA degradation assay of intercellular washing fluids from pistils. (e) RNase activities of intercellular washing fluids from pistils. Data from independent experiments are shown (means ± SDs; n = 6; NS, not significant, *P* > .05; Mann-Whitney *U* test).

To examine the functions of pistils and pollen grains in transgenic tobacco plants, wild-type and transgenic tobacco pistils were pollinated with transgenic and wild-type pollen grains, respectively. As a result, both wild-type and transgenic tobacco plants produced well-developed heterozygous seeds (Figure 4a). To test the possibility of SI among transgenic tobacco plants, one transgenic pistil was pollinated using pollen grains from another transgenic tobacco plant. Unlike the capsules produced by natural self-pollination, these transgenic capsules contained well-developed seeds (Figure 4a). In contrast, when pistils were pollinated using pollen grains from the same flowers of transgenic tobacco plants, all the self-pollinated transgenic tobacco plants developed small abnormal seeds without any well-developed seeds (Figure 4a).

## Characteristic of SI in transgenic tobacco plants

To explore the possible causes of small seeds in the self-pollinated transgenic tobacco plants, the growth of pollen tubes in styles was observed. For the wild-type control, pollen tubes were observed from the style to the ovary, indicating normal pollen-tube growth after pollination (Figure 4b). However, for the self-pollinated transgenic tobacco plants, although pollen grains germinated on the stigma, pollen tubes were not detected below the middle region of the style (Figure 4c). This indicated arrested pollen-tube growth, which caused the SI-like phenotypes in transgenic tobacco plants.

The female determinant for Solanaceae-type SI is a ribonuclease.^14^ Thus, the RNase activities of wild-type and transgenic styles were measured and compared. However, neither RNA degradation assays nor measurements of RNase activities in style extracts showed any differences between wild-type and transgenic styles (Figure 4d and e).

To explore whether this transgene method, which could change a self-compatibility (SC) tobacco plant to an SI-like plant, works in other plant species, transgenic *Arabidopsis* plants overexpressing *GbDFR6* were constructed and analyzed. Although transgenic *Arabidopsis* plants overexpressing *GbDFR6* showed shorter silique lengths (Fig S2A and B), they did not exhibit SI-like phenotypes (Fig S2C and D). This indicated that this method cannot be used universally among plants to convert an SC plant to an SI-like plant.

## Discussion

Flavonoids are a large group of plant secondary metabolites that play multiple roles in plant growth and development, including biotic or abiotic stress responses, pigmentation, nodulation and auxin-transport regulation.^15,16^ Flavonoids are also essential for male fertility in some species. Defects in the flavonoid synthetic pathway lead to reductions in pollen germination rates and pollen-tube lengths.^17,18^ In our experiment, the pollen viability was normal, and the fertility defect in transgenic tobacco might be caused by SI. Considering the key role of DFR in plant flavonoid biosynthetic pathways,^2^ our result indicates a novel role of flavonoids (or anthocyanins) in plant SI.

In our experiment, naturally self-pollinated flowers of transgenic tobacco plants exhibited small/large seed patterns in capsules (Figure 1b). In contrast, restricted self-pollinated flowers on transgenic tobacco plants produced only small seeds, without any well-developed seeds (Figure 4a). Furthermore, the genotype segregation ratios of seedlings, developed from the large seeds, varied among different repetitions (data not shown). To solve this contradiction, we propose that the limited number of large well-developed seeds did not result from self pollination, but were pollinated by nearby flowers. Thus, the genotype-segregation ratio of large seeds may be dependent on the pollen genotypes of nearby flowers.

A developing seed contains three genetically distinct structures: the embryo, the endosperm, and the seed coat. Unlike the fertilization products of embryo and endosperm, the seed coat originates from the ovule integuments and is purely of maternal origin.^19^ Although fertilization is necessary to initiate seed development in most plant species, apomicts have evolved mechanisms allowing seed formation independently of fertilization, and hormone auxin is considered as a molecular trigger of fertilization-independent seed development.^20^ Specifically, seed coat development is driven by auxin, and exogenous application of auxin to unfertilized ovules is sufficient to initiate autonomous development of the seed coat in *Arabidopsis*.^21^ In our research, there were no significant differences in the auxin contents between wild-type and transgenic early developing seeds (Figure 1j). This supported the observation of seed coat development in unfertilized transgenic seeds.

It was proposed that the seed coat development is triggered by a signal delivered by the pollen (the most likely hypothesis is the high levels of auxin in the pollen tube) and sustained by auxin formed in the endosperm.^20^ Furthermore, the auxin biosynthesis in the endosperm are controlled by paternally expressed imprinted genes.^22^ In our research, the pollen-tube growth was arrested in the transgenic tobacco styles (Figure 4c), preventing the delivery of paternal genome to ovules. Thus, the continuous supply of auxin could not be achieved, leaving the smaller-sized seeds in transgenic tobacco plants.

The reversal of SC to SI in transgenic tobacco plants indicates the reactivation of female or male determinants of SI. Although the RNase activities were not altered in transgenic plants, the involvement of S-RNase in SC to IC reversion cannot be ruled out. Because there are different classes of secreted RNases involved in RNA homeostasis, the activity of RNase examined in our experiment may mask the changes in S-RNase activities.^23^ On the other hand, the SI phenotype of transgenic tobacco plants might also be caused by the reactivation of a male determinant. *Nicotiana alata* is a diploid SI plant, whereas the related *Nicotiana tabacum*, is an allotetraploid SC plant.^24^ Although, the molecular mechanism causing *N. tabacum* to be an SC plant is still unclear, it is possible that during *N. tabacum* evolution, *S*-locus duplication occurred, which created two recombining haplotypes within the same genome, allowing SLFs from one haplotype to detoxify S-RNases from the other.^7,25^ In the transgenic tobacco (*N. tabacum*), the duplicated *S*-loci might be inactivated by the products of *GbDFR6*, causing a return to SI in transgenic tobacco.^25^ Because selfed wild-type pollen-tube (separate from meiosis) growth is still arrested in heterozygous transgenic styles, we hypothesized that the inactivation of duplicated *S*-loci occurs before the completion of meiosis.

SI is not representative of only one mechanism but encompasses a collection of divergent systems. Various and distinct types of SI mechanisms have been discovered, including the Solanaceae-type SI and Brassicaceae-type SI.^8^ Tobacco belongs to Solanaceae, whereas *Arabidopsis* belongs to Brassicaceae. Thus, it is reasonable that due to the different mechanisms causing SI, the overexpression of *DFR6* converts only tobacco, but not *Arabidopsis*, to a SI-like plant.

Only transgenic tobacco plants overexpressing *GbDFR6*, but not other *GbDFR* genes, exhibit SI-like phenotypes. This indicates the divergence of gene functions in *GbDFR* gene family members, which is common in the evolution of plant secondary metabolic pathways.^26^ Coincidently, the preferential expression of *GbDFR6* in the ginkgo seed also indicates its specific role in the developing ginkgo seed.^4^ As a dioecious plant, SI mechanism should have not evolved in ginkgo. Thus, although preferentially expressed in the reproductive organ, the function of GbDFR6 in ginkgo is still to be revealed.

To achieve higher yields, most crops are SC plants. However, in the cross-breeding process, the maternal parent should be SI, so that large-scaled crossings are possible. In this report, overexpressing *GbDFR6* converted the SC tobacco plants to SI-like plants. Although the molecular mechanism remains unclear, this may provide a valuable method for creating SI tobacco plants in future breeding experiments.

## Supplementary Material

Supplemental Material
