# Supplementary figures and images for: Overexpression of the Ginkgo biloba dihydroflavonol 4-reductase gene GbDFR6 results in the self-incompatibility-like phenotypes in transgenic tobacco
Source: Plant Signal Behav. 2023 Jan 11;18(1):2163339. doi: 10.1080/15592324.2022.2163339 (PMC9839370; doi:10.1080/15592324.2022.2163339)

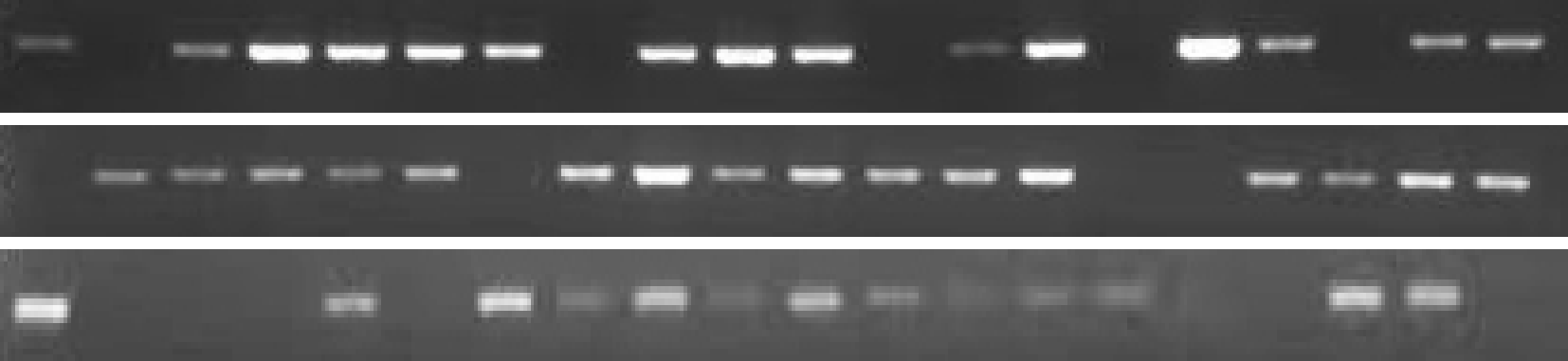

Supplement: Supplemental Material [file KPSB_A_2163339_SM7298.zip › Figure S1.tif]

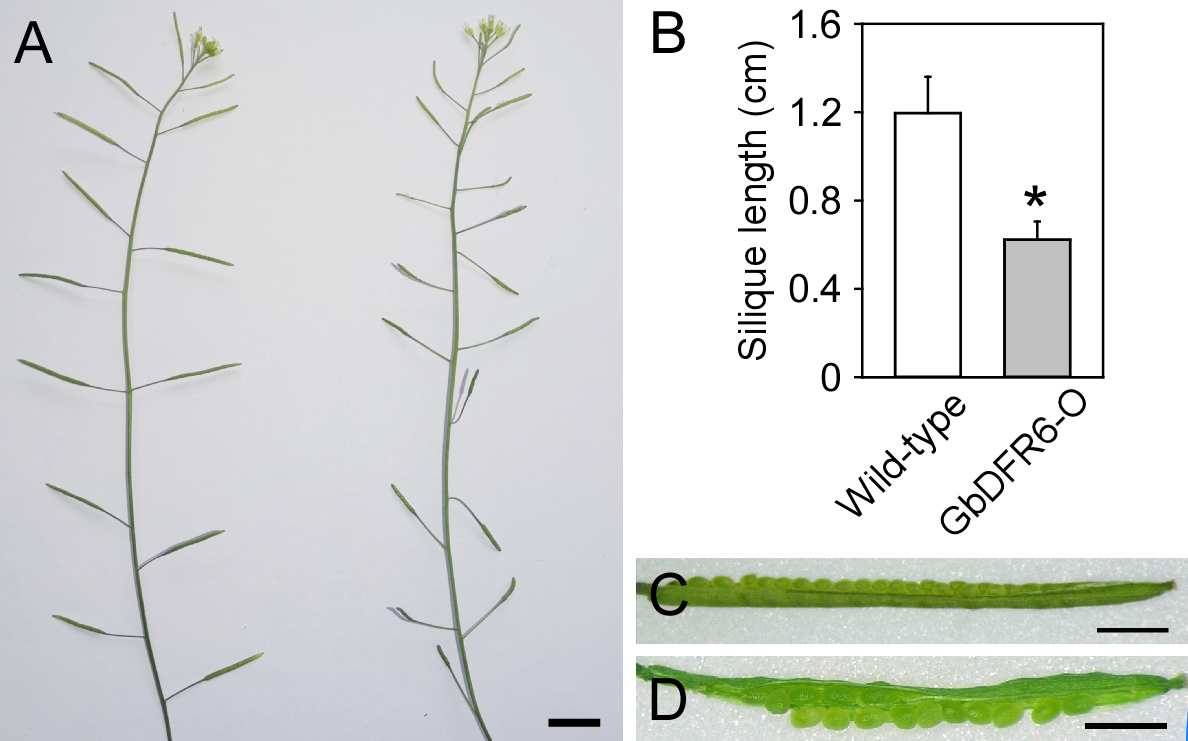

Supplement: Supplemental Material [file KPSB_A_2163339_SM7298.zip › Figure S2.tif]
